# Sharing Mono-Institutional Experience of Treating Pancreatic Cancer with Stereotactic Body Radiation Therapy (SBRT)

**DOI:** 10.3390/curroncol31100446

**Published:** 2024-10-04

**Authors:** Asmara Waheed, Shannah Murland, Eugene Yip, Amr Heikal, Sunita Ghosh, Aswin Abraham, Kim Paulson, Keith Tankel, Nawaid Usmani, Diane Severin, Clarence Wong, Kurian Joseph

**Affiliations:** 1 Division of Radiation Oncology, Department of Oncology, University of Alberta & Cross Cancer Institute, Edmonton, AB T6G 1Z2, Canadaaswin.abraham@ahs.ca (A.A.); kim.paulson@ahs.ca (K.P.); keith.tankel@ahs.ca (K.T.); nawaid.usmani@ahs.ca (N.U.); diane.severin@ahs.ca (D.S.); 2 Department of Radiation Therapy, Cross Cancer Institute, Edmonton, AB T6G 1Z2, Canada; shannah.murland@ahs.ca; 3 Division of Medical Physics, Department of Oncology, University of Alberta, Edmonton, AB T6G 1Z2, Canada; eugene.yip@ahs.ca (E.Y.); amr.heikal@ahs.ca (A.H.); 4 Division of Medical Oncology, Department of Oncology, University of Alberta & Cross Cancer Institute, Edmonton, AB T6G 1Z2, Canada; sghosh1@ualberta.ca; 5 Division of Gastroenterology, Department of Medicine, University of Alberta, Edmonton, AB T6G 1Z2, Canada; clarence.wong@ahs.ca

**Keywords:** pancreatic cancer, stereotactic body radiation therapy (SBRT), unresectable

## Abstract

Background: Stereotactic body radiotherapy (SBRT) is an evolving treatment for the local management of pancreatic cancer (PC). The main purpose of this study is to report our initial experience in terms of local control (LC) and toxicity for PC patients treated with SBRT. Methods: We conducted a retrospective review of patients treated with SBRT using abdominal compression (AC) or an end-expiratory breath-holding (EEBH) technique. The median prescribed dose was 35 Gy, delivered in five fractions. Toxicities were recorded using Common Terminology Criteria for Adverse Events (CTCAE) v5.0, and survival was estimated using the Kaplan–Meier method. Results: From 2017 to 2023, 17 PC patients were offered SBRT. Their median age was 69 years. The median follow-up from the date of diagnosis was 22.37 months. The overall survival (OS) was 94% at 1 year and 60.9% at 2 years. The progression-free survival (PFS) was 63.1% at 6 months and 56.1% at 9 months. The median OS was 26.3 months, and the median PFS was 20.6 months. The 6-month and 1-year LC rates were 71% and 50.8%, respectively. Conclusion: We are successful in implementing the SBRT program at our centre. SBRT appears to be a promising treatment option for achieving LC with limited acute toxicities.

## 1. Introduction

Pancreatic cancer (PC) accounts for 1.8% of all cancers and is one of the most aggressive, with a 5-year overall survival rate [1] of 6% and a median overall survival (OS) [2] of up to 13.6 months.

The management of pancreatic cancer continues to be a challenge. At diagnosis, only 20% of patients have resectable tumours, 40% have unresectable tumours, and 40% present with occult or evident metastatic disease [3]. Very few patients are diagnosed with early disease, but those treated with curative surgical resection have shown improved outcomes [4]. In patients with locally advanced or borderline resectable pancreatic cancer that involves critical abdominal vasculature, an upfront surgical resection may be challenging or sometimes impossible [5]. In these patients, neoadjuvant treatment regimens have been introduced to downsize and downstage the tumour to enable resection [6]. In the majority of patients, gemcitabine or FOLFIRINOX (5-fluorouracil + leucovorin + irinotecan + oxaliplatin)-based chemotherapy regimens are used as an initial therapy, with or without radiation therapy [7,8]. The idea behind adding radiation therapy as a part of a neoadjuvant treatment regimen is to maximize local control and improve resectability [9]. Because pancreatic cancer is an intrinsically radioresistant tumour, a higher biologically effective dose (>100 Gy) is likely needed for effective tumour ablation [10].

Previously, external beam radiotherapy was delivered by three-dimensional conformal or intensity-modulated radiation therapy techniques. The ability of these techniques is limited to large-field radiation, so conventional fractionation was used to reduce toxicity. With the advent of stereotactic body radiation therapy (SBRT), maximum ablative radiation doses can be delivered to pancreatic tumours using hypofractionated regimens. SBRT not only produces highly cytotoxic ablative biological effects but also enhances an anti-tumour immune response, which can lead to the destruction of metastases far from the irradiated lesion. However, its abscopal effect is not clinically substantiated [11]. It is generally tolerated better than conventional radiotherapy due to the use of smaller target volumes, but it is a technically more complex treatment than conventional radiotherapy.

A phase II study by Herman et al. has shown that the use of fractionated SBRT (33 Gy in 5 fractions) with gemcitabine to treat locally advanced pancreatic cancer has resulted in high rates of tumour control with low rates of serious adverse events [12]. Recent data favour the use of SBRT in neoadjuvant settings due to its beneficial effect on delaying local progression and improving rates of R0 resection and local control in locally advanced borderline resectable pancreatic cancer [12,13].

SBRT is a challenging procedure requiring careful patient selection and subspecialized expertise. Consequently, for the effective and safe delivery of ablative doses through techniques such as SBRT, a number of factors should be taken into account, including anatomic considerations, the coverage of nodal regions and vessels, dose heterogeneity, organ motion management, and technology use (e.g., image guidance).

This study reports our institutional experience and the clinical outcomes of pancreatic cancer patients treated with SBRT.

## 2. Materials and Methods

Our centre started our pancreatic SBRT program in 2017. Approval was obtained from the Alberta Research Ethics Committee Review Board to retrospectively review the procedures and outcomes of pancreatic cancer patients treated with SBRT at our institution during the period from April 2017 to September 2023.

### 2.1. Study Design and Data Collection

The data of patients with biopsy-proven pancreatic cancer treated with SBRT were collected. Pretreatment staging work-up, including computed tomography (CT) of the chest, abdomen, and pelvis; measurement of CA 19-9 levels; and magnetic resonance imaging (MRI) according to pancreatic protocol, was performed to determine resectability. All the patients were inoperable and initially treated with neoadjuvant chemotherapy, then subsequently evaluated by a hepatobiliary surgeon and deemed unresectable or medically inoperable. All the patients were evaluated by a multidisciplinary tumour board and deemed appropriate for treatment with SBRT.

### 2.2. SBRT Details

Preparation for SBRT planning includes endoscopic insertion of MRI-compatible gold fiducial markers into the target lesion in the pancreas, enabling tumour tracking during treatment delivery.

Once the patient was positioned in a custom immobilisation device (VacQfix bags on a CIVCO board, CQ Medical, Avondale, PA) in the treatment position, a fluoroscopy session on the treatment unit was performed to visualize fiducial motion with respiration. The best means of breathing motion management was chosen, with the goal of minimizing target motion. End-expiration breath-hold (EEBH) was recommended if the patient could hold their breath for at least 25 seconds and the breath hold was stable and reproducible. If the EEBH was not of adequate length or deemed irreproducible, abdominal compression (AC) was available as a second option to reduce fiducial/diaphragmatic motion to 5–7 mm. If the residual motion with abdominal compression remains too large, a respiratory gating technique can be used whereby the patient breathes normally and treatment is only delivered during the expiratory phase of the respiratory cycle.

### 2.3. CT Simulation

Each patient underwent CT simulation with oral and intravenous contrast, with a breath-hold 3-dimensional CT (3DCT) for EEBH patients and a 4-dimensional CT (4DCT) for AC and gated patients.

An MRI scan was obtained in the treatment position at the time of simulation and was fused to the CT simulation images to assist in target delineation. All treatment planning was performed using the Eclipse treatment planning system (Varian Medical Systems, Palo Alto, CA, USA, version 15.6).

### 2.4. Target Volumes

Gross tumour volume (GTV) was defined as the gross tumour only and there was no elective nodal regional coverage included in the target. There was no internal target volume (ITV) created for patients treated with EEBH as there was no respiratory motion present. For non-EEBH patients with 4DCTs, the ITV was defined by the union of contours generated in two breathing phases representing the maximum motion extent of the tumour in the treatment window. The planning target volume (PTV) was generated by adding a 0.5 cm uniform volumetric expansion around the GTV or ITV. The relevant organs at risk (OARs) (Table 1) were the liver, duodenum, stomach, small and large bowels, kidneys, and spinal cord. For patients with 4DCT data, contouring of OARs was performed on the averaged 4DCT breathing phases in the treatment window.

All targets and dose constraints were peer-reviewed by a second radiation oncologist (RO) before and after planning. The prescription dose was 35–50 Gy in five fractions, and the dose constraints used are given in Table 1. The required target coverage for the PTV was that 95% of the PTV should receive a minimum of 100% of the prescribed dose, and the maximum dose should be limited to <150% and be within the GTV/ITV. The SBRT plan optimization was performed with a volumetric modulated arc (VMAT) technique. The radiation plan was prospectively evaluated by a medical physicist for quality assurance (QA) and reviewed at a gastrointestinal (GI) RO QA meeting for final approval before treatment delivery.

Each patient was booked for a mock setup appointment prior to the first treatment to assess immobilization and motion management, and this appointment was attended by a physician, a medical physicist, and a member of the dosimetry team. Daily pre-treatment imaging consisted of cone beam CT (CBCT) that was initially matched to fiducials and then verified against soft tissue anatomy. Once the match was deemed acceptable by the RO, fluoroscopic images were acquired to ensure that the position of the fiducials was within tolerance. Treatment was delivered on non-consecutive days on a TrueBeam STx (Varian Medical Systems Inc., Palo Alto, CA, USA) linear accelerator (LINAC). During delivery, both an external surrogate (respiratory block on the patient’s abdomen tracked by infrared cameras) and internal surrogates (fiducials imaged with kV images every few seconds as part of the Varian’s Auto Beam Hold system) were used to ensure treatment accuracy. 

### 2.5. Follow Up

Patients were seen by their RO during radiation treatment and then evaluated every 3 months thereafter. They were evaluated for local control and progression based on RECIST criteria [14], and acute and late toxicities were scored according to Common Terminology Criteria for Adverse Events (CTCAE) V5.0 [15].

### 2.6. Statistical Methods

Descriptive statistics were presented for the study variables. Mean and standard deviation (SD) were reported for continuous variables, and frequency and proportions were reported for categorical variables. Overall survival (OS) was calculated from the date of diagnosis as well as the date of the first radiation treatment to the date of death due to any cause and patients alive at the end of the study were censored. Progression-free survival (PFS) was calculated from the start of radiation to the date of first documented progression, and patients who did not progress were censored. Kaplan–Meier estimates were reported as the median and the corresponding 95% confidence intervals. All statistical tests were conducted using SPSS (IBM Corp. Released 2022. IBM SPSS Statistics for Windows, Version 29.0. Armonk, NY, USA: IBM Corp) version 29 software.

## 3. Results

### 3.1. Patient and Treatment Characteristics

From April 2017 to September 2023, 17 patients with PC were offered SBRT. All patients had locally advanced unresectable disease invading vessels or adjacent structures except two who had borderline resectable disease at presentation. Patients’ characteristics are shown in Table 2. The median age was 69 years (range: 47–85), and the median follow-up from the date of diagnosis was 22.37 months (range: 7.5–81.1). All patients completed follow-up and there were only four patients (23%) alive at the time of analysis. The main indication for treatment was palliation for LC, and other indications were oligometastatic disease, additional treatment post-chemotherapy for conversion into resectable disease, or salvage therapy after surgery.

Most patients (88%) had an ECOG performance status of 0–1 at the time of diagnosis. An average of ten cycles of induction chemotherapy, mainly FOLFIRINOX (40% of patients), was offered. The remaining four patients (24.7%) received either gemcitabine, gemcitabine plus capecitabine, or gemcitabine with nab-paclitaxel combination chemotherapy. Surgery was performed in one patient (5.9%) as primary therapy, while SBRT was offered in three patients (17.6%) without any prior therapy. Two patients with diagnoses of melanoma and insulinoma were treated with nivolumab and octreotide, respectively.

Five patients presented with obstructive jaundice at the time of presentation and required a hepatopancreatoduodectomy (HPD) stent. All patients completed the full SBRT course and had no nodal disease at the time of SBRT.

### 3.2. Dosimetric Features

**Figure 1:**
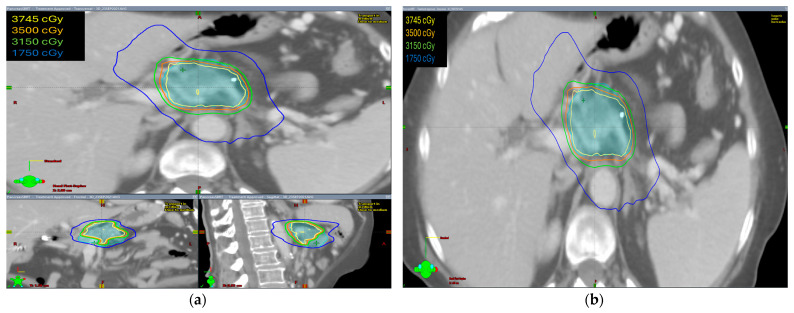
The isodose distribution using abdominal compression technique (**a**) axial, coronal, and sagittal views (**b**) axial view.

**Figure 2:**
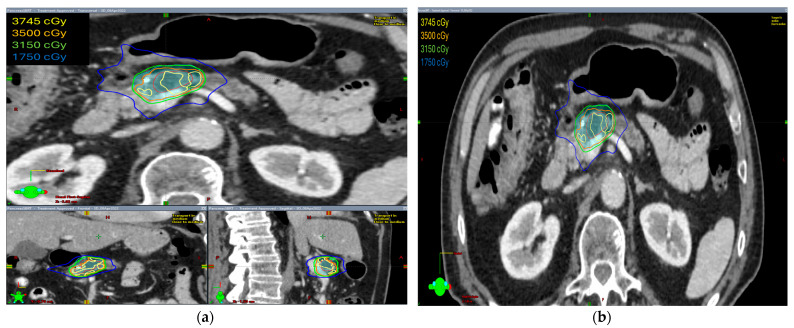
The isodose distribution using the breath-hold technique (**a**) axial, coronal, and sagittal views (**b**) axial view.

The radiation prescription dose was 35 Gy in five fractions in ten patients (58.8%). In five patients, the dose prescription was reduced to 30–33 Gy in five fractions and two patients received 40–50 Gy in five fractions. Median GTV volume was 28.60 cc (range 4.9–145.8 cc), median ITV was 29.15 cc (range 5.0–149.7 cc), and median PTV was 75.1 cc (range 19.9–255.3 cc). The isodose distribution for both abdominal compression and breath-hold technique is shown in Figure 1 and Figure 2, respectively.

The isodose distribution curves for target coverage and normal tissue dosing were evaluated. We found that the ITV was generated only in patients with abdominal compression to encompass the internal organ motion during treatment. Hence, PTV volumes were larger in these patients as compared with patients treated with breath-hold in which no ITV was drawn and smaller PTV volumes were irradiated.

Table 3 summarizes all the dosimetric characteristics of the study patients, and Table 4 represents the dosimetric features of the organs at risk (OARs) derived from the analysis of the dose volume histograms (DVHs). We found that all of the OARs were within the acceptable in-house protocol tolerance limit. The mean doses to bilateral kidneys were less than 10 Gy, and for the small bowel, stomach, and duodenum, the volume that received 33 Gy remained less than 0.5 cc. The spinal cord maximum tolerance limit recorded as dmax was less than 18 Gy.

### 3.3. Overall Survival and Progression-Free Survival

**Figure 3:**
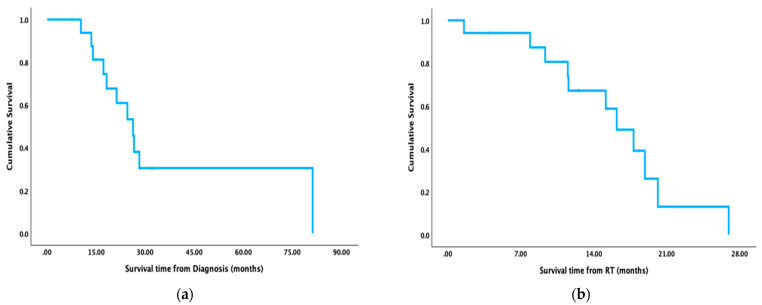
Kaplan–Meier curves (**a**) Overall survival (OS) calculated from the date of diagnosis (**b**) overall survival calculated from the date radiation started.

The overall survival (OS) calculated from the date of diagnosis was 94% at one year, 60.9% at two years, and 30.5% at three years (Figure 3a). The overall survival calculated from the date the radiation started was 67.2% at one year and 13.1% at two years (Figure 3b). The progression-free survival (PFS) was 63.1% at six months, 56.1% at nine months, and 56.1% at one year. The median OS, as calculated from the diagnosis, was 26.3 months; the median OS, as calculated from the start of radiation, was 16.2 months, and the median PFS was 20.6 months. The six-month and one-year local control rates were 71% and 50.8%, respectively.

### 3.4. Comparison of Treatment Time with Abdominal Compression versus Breath-Hold Technique

Ten patients (58.8%) were treated with an AC technique, while seven patients (41.2%) were treated with an EEBH technique. The median time required for delivering SBRT using abdominal compression was 4.6 minutes compared to 8.8 minutes for the EEBH technique. This time includes dose delivery with appropriate imaging during treatment. But set-up and pre-treatment imaging add considerable time, and these results are also shown below (median 17.7 minutes for AC versus 20.3 minutes for EEBH). Table 5 depicts the time taken to treat patients with AC versus EEBH motion management techniques and planning target volumes. Although more time is required for treating patients with the EEBH technique, it resulted in comparatively smaller target volumes being treated (49 versus 52 cc).

### 3.5. Toxicity

No radiation-related toxicities were reported during RT or at three and six months follow-up. Most patients had grade 1 fatigue during radiation that required no interventions. Two patients (11.7%) developed grade I/II gastrointestinal bleeding (GI) due to progression at six and nine months, respectively, while one patient (5.9%) developed a lower GI bleed at six months. A total of eleven deaths were reported, three of which were caused by disease progression. Four patients died from failure to thrive, and another four died of unrelated causes such as sepsis and cardiogenic shock.

## 4. Discussion

In patients with locally advanced unresectable non-metastatic pancreatic cancer, the treatment is controversial and, unfortunately, the prognosis remains poor due to most patients being judged as inoperable at diagnosis. In pancreatic cancer, surgical resection is the only treatment option with the potential for long-term survival and cure. Even after complete curative resection, the expected 5-year survival remains 20% due to higher rates of local recurrence [5]. Studies have shown that a regimen of chemotherapy alone can reduce the incidence of distant metastases in patients with localized disease, with a median survival range of 9–14 months, but it may have little impact on local control [16]. Therefore, utilization of local ablative techniques like SBRT is important in the subset of patients with unresectable, locally advanced pancreatic cancer. However, its exact role and the optimal sequencing in the context of other treatments are still unknown. The published data demonstrate that SBRT can lead to improved local control, provide a beneficial effect on quality of life, and can reduce the risk of severe complications such as gastric and biliary obstruction, bleeding, and chronic pain, but there are no phase III clinical trial data that can confirm the findings. Therefore, the majority of treatments are based on clinical experience derived from a single institution’s series. In this article, we have highlighted the outcomes of real-world locally advanced pancreatic cancer patients treated with SBRT at our institution, which can add to the available body of evidence.

On the contrary, the use of chemoradiation to attempt to convert tumours to resectable ones is controversial. The reported local progression rates with conventionally fractionated RT are 40–55% [17,18]. The best evidence to date remains that collected by LAP-07, an open-label randomized phase III trial that assigned patients with locally advanced PC (n = 442) to induction chemotherapy for 4 months. In the absence of disease progression, patients (n = 269 patients) were randomly assigned to receive either concurrent chemoradiation (54 Gy + capecitabine) or more chemotherapy for two months. The results showed no benefit of radiation (median OS 15.2 v 16.5 months, HR 1.03, *p* = 0.83) [2].

Considering the poor survival of locally advanced PC patients, much of the research from the last decade has focused on improving local control. Due to the conflicting results of conventional RT for locally advanced PC [19,20], several studies have investigated the feasibility and efficacy of stereotactic radiation techniques, which permit the precise application of high-dose radiation to a limited target volume, thereby reducing the radiation dose to adjacent healthy tissue and subsequently minimizing toxicity. Our study has reported six-month and one-year local control rates of 71% and 50.8%, respectively, which is relatively low as compared to other series. An overview of the efficacy of SBRT for locally advanced PC is given in Table 6. A higher rate of local control was observed in patients with a higher median dose and an increased number of fractions.

Although many studies are completed using small cohorts, and patient characteristics are not homogenous, the impact of SBRT on improving local control is very significant, with a success rate of 60–90% [21,22,23,24,25,26,27,28]. On the one hand, the beneficial effect of SBRT on local control has been proven, but on the other, the data on the late toxicity of SBRT have shown an increased risk of gastroduodenal toxicity.

In our study, the majority of patients with locally advanced unresectable adenocarcinoma of the pancreas were treated with the prescription of 35 Gy in 5 fractions. This is in accordance with the data published by Chuong et al., who treated 73 patients with locally advanced unresectable PC with a median dose of 35 Gy in 5 fractions (median high BED^10^ of 59.5 Gy) and had a local control rate of 81% at one year [9]. However, the RT dose was slightly lower (30–33 Gy) in five patients and marginally higher in two patients (45 Gy), which is in agreement with Song et al., who treated 59 patients with locally advanced pancreatic cancer with 45 Gy in 5 fractions (median BED^10^ of 85.5 Gy) and reported freedom from local progression of 90.8% at one year [29]. Of note, radiation dose prescription was kept lower (30–33 Gy) in patients with tumours in closer proximity to the duodenum and bowel loops.

In our cohort, we had eleven patients who received prior chemotherapy before SBRT, but the remaining ones had either surgery, immunotherapy, or radiation upfront, and we did not notice any difference in local control. Additionally, we found the median overall survival calculated from diagnosis was 26.3 months and from the start of radiation, it was 16 months, which is comparable with the results reported by several studies in the literature [24,25,28]. Despite the negligible effect of local control on overall survival benefits, it still has a positive impact on reducing the risk of bowel and biliary obstruction and other morbid complications, as we did not find any of these complications in our patient population. Furthermore, the biologically equivalent dose of SBRT is higher than the conventionally fractionated external beam radiation treatment for both tumour and normal tissue; therefore, we kept them within tolerance for the organs at risk.

In general, reducing the target volume is the primary goal of pancreatic SBRT. Studies have shown that larger target volumes are associated with higher rates of gastrointestinal toxicity, so if we control the internal organ motion of the tumour with abdominal compression or the end-exhale breath-hold technique, gastrointestinal toxicity can be reduced. We found the breath-hold technique utilized smaller PTV margins as compared with abdominal compression, which required an ITV and could cause discomfort to the patient. Abdominal compression also had the potential to push the OARs closer to the target volume. Although the treatment times are comparatively longer for breath-hold techniques, we still consider it our preferred method of motion management. We found no perforation or grade III GI toxicities, thus confirming the safety of this SBRT regimen. We believe that with higher conformity and lesser target volume margins, we were able to achieve this optimal toxicity profile.

This study has some limitations, including the small sample size and heterogeneity in the patient population in terms of diagnostic stage, dose of SBRT, and use of chemotherapy.

## 5. Conclusions

In conclusion, we were successful in implementing a pancreas SBRT program at our centre. End-exhalation breath-hold was found to be the preferred motion management technique despite longer treatment times. Within the limitations of a relatively small sample size, this study demonstrated that SBRT appears to be a promising treatment option to achieve local control with limited acute toxicities. Future studies involving dose escalation are planned as part of a clinical trial to increase the impact on survival for these patients.

## Figures and Tables

**Table 1:** Dose-volume constraints for OARs.

Organs at Risk	Dose Volume Constraints
Duodenum	V33Gy < 0.5 cc
	V20Gy < 3 cc
	V15Gy < 9 cc
Liver	V10Gy < 70%
Kidneys	Mean < 10 Gy
Spinal Cord	Dmax < 18 Gy
	V8Gy < 1 cc
Small Bowel	V33Gy < 0.5 cc
	V20Gy < 3 cc
	V15Gy < 9 cc
Large Bowel	V35Gy < 0.5 cc
Stomach	V33Gy < 0.5 cc
	V20Gy < 3 cc
	V15Gy < 9 cc

**Table 2:** Summary of patient characteristics.

**Total Patients Number**	17
**Mean Age**	9 (47–85)
**Gender**	
(M:F)	14:3
**Smoking Status**	
Nonsmokers	10 (58.8%)
Smokers	7 (41.2%)
**Pathology**	
Adenocarcinoma	15(88.2%)
Other	2 (11.8%)
**TNM stage**	
IA	2(11.8%)
IB	4(23.5%)
IIB	3(17.7%)
III	8(47.1%)
**Site of Pancreatic Cancer**	
Head	10 (58.8%)
Neck	3(17.6%)
Body	2 (11.8%)
Tail	1(5.9%)
Periampullary	1 (5.9%)
**Fiducial insertion**	
Yes	13(76.5%)
No	4 (23.5%)
**Mean Dose**	35 Gy (23–50)
**Motion Management Technique**	
Abdominal Compression	10 (58.8%)
Exhale Breath-Hold	7 (41.2%)
**Prior Therapy**	
Chemotherapy	11 (64.7%)
Surgery	1 (5.9%)
Radiation therapy	3 (17.6%)
Immunotherapy	2 (11.8%)

**Table 3:** Summary of the DVH analysis of the CTV and PTV for the entire cohort of patients.

Organ	Parameter	Mean ± SD	Range
GTV	Vol (cc)	47.61 ± 46.97	[4.9; 145.8]
	Mean (Gy)	39.13 ± 5.94	[25.8; 49.5]
	Min (Gy)	27.35 ± 5.41	[16.6; 34.50]
	Max (Gy)	45.71 ± 7.79	[29.8; 58.8]
ITV	Vol (cc)	48.43 ± 52.06	[5.0; 149.7]
	Mean (Gy)	36.71 ± 5.59	[25.64; 44.52]
	Min (Gy)	26.02 ± 5.98	[16.55; 34.41]
	Max (Gy)	42.46 ± 6.99	[29.81; 52.40]
PTV	Vol (cc)	97.87 ± 79.33	[19.9; 255.3]
	Mean (Gy)	35.91 ± 4.62	[25.12; 42.23]
	Min (Gy)	18.93 ± 7.55	[8.34; 30.46]
	Max (Gy)	45.17± 7.93	[29.81; 58.87]

**Table 4:** Summary of the DVH analysis of the OARs for the entire cohort of patients.

Organ	Parameter	Objective	Mean ± SD	Range
Left Kidney	Vol (cc)		169.9 ± 41.06	[90.7; 249.5]
	Mean (Gy)		2.71 ± 1.41	[0.79; 5.76]
	Min (Gy)		0.17 ± 0.11	[0.04; 0.5]
	Max (Gy)		10.32 ± 5.13	[3.46; 21.95]
	V15Gy (%)		0.14 ± 0.38	[0; 1.19]
	D1cc (Gy)		8.84 ± 4.31	[2.9; 17.2]
Right Kidney	Vol (cc)		160.5 ± 44.44	[90.5; 246.5]
	Mean (Gy)		4.05 ± 2.74	[1.36; 9.92]
	Min (Gy)		0.29 ± 0.44	[0.07; 1.90]
	Max (Gy)		13.39 ± 5.70	[4.77;25.79]
	V15Gy (%)		1.20 ± 3.24	[0; 12.85]
	D1cc (Gy)		11.29 ± 4.77	[4.3; 21.7]
Small Bowel	Vol (cc)		744.2 ± 505.3	[120.6; 1762.1]
	Mean (Gy)		3.45 ± 2.29	[0.75; 7.82]
	Min (Gy)		0.18 ± 0.19	[0.01;0.78]
	Max (Gy)		26.92± 28.04	[12.47; 36.87]
	D3cc (Gy)		19.23 ± 6.77	[7.2; 28.4]
	V33Gy (cc)	V33Gy < 0.5 cc	0.02 ± 0.06	[0; 0.19]
	V20Gy (cc)	V20Gy < 3 cc	6.09 ± 10.04	[0; 40.07]
	V15Gy (cc)	V15Gy < 9 cc	17.84 ± 29.58	[0; 122.7]
Large Bowel	Vol (cc)		728.5 ± 399.5	[78.8; 1514.7]
	Mean (Gy)		3.95 ± 1.91	[0.69; 8.49]
	Min (Gy)		0.09 ± 0.07	[0.01; 0.28]
	Max (Gy)		24.85 ± 10.35	[7.17; 46.35]
	D3cc (Gy)		18.27 ± 7.27	[5.6; 32.5]
	V35Gy (cc)	V35Gy < 0.5 cc	0.09 ± 0.39	[0; 1.61]
Liver	Vol (cc)		1516.8 ± 523.5	[176.9; 2255.2]
	Mean (Gy)		1.86 ± 1.57	[0.23; 5.42]
	Min (Gy)		0.07 ± 0.05	[0.02; 0.2]
	Max (Gy)		22.18 ± 12.51	[6.67; 43.62]
	V21Gy (cc)		6.78 ± 16.36	[0; 61.82]
	V_spare_ (V_tot_-V21Gy) (cc)		1510 ± 527.2	[176.89; 2255.2]
	V10Gy (%)	V10Gy < 70%	3.85 ± 5.75	[0; 19.16]
Stomach	Vol (cc)		274.4 ± 157.9	[22.3; 698]
	Mean (Gy)		1.97 ± 2.46	[0.19; 7.66]
	Min (Gy)		0.16 ± 0.10	[0.04; 0.44]
	Max (Gy)		12.30 ± 12.34	[0.46; 30.99]
	D3cc (Gy)		8.29 ± 9.24	[0.4; 25.2]
	V33Gy (cc)	V33Gy < 0.5 cc	0 ± 0	[0; 0]
	V20Gy (cc)	V20Gy < 3 cc	1.16 ± 2.76	[0; 10.87]
	V15Gy (cc)	V15Gy < 9 cc	5.43 ± 10.29	[0; 37.86]
Duodenum	Vol (cc)		5.15 ± 49.66	[17.3; 196.5]
	Mean (Gy)		8.22 ± 4.89	[2.12; 19.87]
	Min (Gy)		0.89 ± 1.09	[0.1; 4.08]
	Max (Gy)		30.39± 8.12	[7.51; 42.22]
	D1cc (Gy)		23.84 ± 8.4	[5.5; 31.3]
	V33Gy (cc)	V33Gy < 0.5 cc	0.07 ± 0.14	[0; 0.55]
	V20Gy (cc)	V20Gy < 3 cc	9.72 ± 14.04	[0; 39.46]
	V15Gy (cc)	V15Gy < 9 cc	15.42 ± 18.25	[0; 55.45]
Spinal Cord	Vol (cc)		32.68 ± 13.29	[15.1; 71.6]
	Mean (Gy)		2.06 ± 1.27	[0.58; 5.19]
	Min (Gy)		0.08 ± 0.09	[0; 0.37]
	Max (Gy)	<18 Gy	8.03 ± 3.38	[2.39; 16.02]
	D1cc (Gy)		6.58 ± 2.73	[2.0; 13.7]
	V8Gy (cc)	V8Gy < 1 cc	6.79 ± 4.44	[0; 14.64]
Both Kidneys	Vol (cc)		330.5 ± 81.05	[181.2; 496]
	Mean (Gy)	<10 Gy	3.32 ± 1.77	[1.06; 7.11]
	Min (Gy)		0.15 ± 0.10	[0.04; 0.49]
	Max (Gy)		15.21 ± 5.12	[7.48; 25.79]

**Table 5:** Comparison of time, dose, and treatment volume between two different motion management techniques.

Abdominal Compression					End-Exhale Breath-Hold				
Time (min)	Delivery	Total	PTV		Time (min)	Delivery	Total	PTV	
Median	4.60	17.67	Volume (cc)	52	Median	8.78	20.28	Volume (cc)	49
Mean	4.75	15.89	Mean dose (Gy)	20.3	Mean	9.47	23.17	Mean dose (Gy)	16.9
Min	2.53	8.03	Min dose (Gy)	10.8	Min	4.32	11.40	Min dose (Gy)	8.8
Max	10.68	29.38	Max dose (Gy)	24.9	Max	22.1	59.50	Max dose (Gy)	21.8

**Table 6:** Efficacy of stereotactic body radiotherapy (SBRT) for locally advanced pancreatic cancer (PC).

Reference	Design	No. of Patients	Median Dose (Gy)	Fractions	Median FU (Months)	Local Control (%)	Median OS (Months)
Schellenberg [21]	Prospective	20	25	1	30	94	11.8
Polistina [22]	Prospective	23	30	3	9	57	10.6
Chang [23]	Retrospective	77	25	1	6	84	11.4
Mahadevan [24]	Retrospective	39	24–36	3	21	85	20
Didolkar [25]	Prospective	85	15–30	3	8	91.7	18.6
Koong [26]	Prospective	15	15–25	1	-	77	11
Rwigema [27]	Retrospective	71	18–25	1	12.7	64.8	10.3
Tozzi [28]	Prospective	30	36–45	6	11	96	19.5

## Data Availability

The datasets presented in this article are not readily available because of patient confidentiality. Requests to access the datasets should be directed to the corresponding author.

## References

[1] Siegel R.L., Miller K.D., Jemal A. (2018). Cancer statistics, 2018. CA Cancer J. Clin..

[2] Hammel P., Huguet F., van Laethem J.L., Goldstein D., Glimelius B., Artru P., Borbath I., Bouché O., Shannon J., André T. (2016). Effect of chemoradiotherapy vs chemotherapy on survival in patients with locally advanced pan-creatic cancer controlled after 4 months of gemcitabine with or without erlotinib: The LAP07 randomized clinical trial. JAMA.

[3] Ducreux M., Cuhna A.S., Caramella C., Hollebecque A., Burtin P., Goéré D., Seufferlein T., Haustermans K., Van Laethem J.L., Conroy T. (2015). Cancer of the pancreas: ESMO Clinical Practice Guidelines for diagnosis, treatment and follow-up. Ann. Oncol..

[4] Chakraborty S., Singh S. (2013). Surgical resection improves survival in pancreatic cancer patients without vascular invasion-a population based study. Ann. Gastroenterol. Q. Publ. Hell. Soc. Gastroenterol..

[5] Toesca D.A., Koong A.J., Poultsides G.A., Visser B.C., Haraldsdottir S., Koong A.C., Chang D.T. (2018). Management of Borderline Resectable Pancreatic Cancer. Int. J. Radiat. Oncol..

[6] Kaufmann B., Hartmann D., D’haese J.G., Stupakov P., Radenkovic D., Gloor B., Friess H. (2018). Neoadjuvant Treatment for Borderline Resectable Pancreatic Ductal Adenocarcinoma. Dig. Surg..

[7] Kim S.S., Nakakura E.K., Wang Z.J., Kim G.E., Corvera C.U., Harris H.W., Kirkwood K.S., Hirose R., Tempero M.A., Ko A.H. (2016). Preoperative FOLFIRINOX for borderline resectable pancreatic cancer: Is radiation necessary in the modern era of chemotherapy?. J. Surg. Oncol..

[8] Jang J.Y., Han Y., Lee H., Kim S.W., Kwon W., Lee K.H., Oh D.Y., Chie E.K., Lee J.M., Heo J.S. (2018). Oncological benefits of neoadjuvant chemoradiation with gemcitabine versus upfront surgery in pa-tients with borderline resectable pancreatic cancer: A prospective, randomized, open-label, multicenter phase 2/3 trial. Ann. Surg..

[9] Chuong M.D., Springett G.M., Freilich J.M., Park C.K., Weber J.M., Mellon E.A., Hodul P.J., Malafa M.P., Meredith K.L., Hoffe S.E. (2013). Stereotactic Body Radiation Therapy for Locally Advanced and Borderline Resectable Pancreatic Cancer Is Effective and Well Tolerated. Int. J. Radiat. Oncol..

[10] Herman J.M., Chang D.T., Goodman K.A., Dholakia A.S., Raman S.P., Hacker-Prietz A., Iacobuzio-Donahue C.A., Griffith M.E., Pawlik T.M., Pai J.S. (2014). Phase 2 multi-institutional trial evaluating gemcitabine and stereotactic body radiotherapy for patients with locally advanced unresectable pancreatic adenocarcinoma. Cancer.

[11] D’andrea M.A., Reddy G.K. (2019). The systemic immunostimulatory effects of radiation therapy producing overall tumor control through the abscopal effect. J. Radiat. Oncol..

[12] Ghaly M., Gogineni E., Saif M.W. (2019). The Evolving Field of Stereotactic Body Radiation Therapy in Pancreatic Cancer. Pancreas-Open J..

[13] Oar A., Lee M., Le H., Hruby G., Dalfsen R., Pryor D., Lee D., Chu J., Holloway L., Briggs A. (2019). Australasian Gastrointestinal Trials Group (AGITG) and Trans-Tasman Radiation Oncology Group (TROG) Guidelines for Pancreatic Stereotactic Body Radiation Therapy (SBRT). Pract. Radiat. Oncol..

[14] Eisenhauer E.A., Therasse P., Bogaerts J., Schwartz L.H., Sargent D., Ford R., Dancey J., Arbuck S., Gwyther S., Mooney M. (2009). New response evaluation criteria in solid tumours: Revised RECIST guideline (version 1.1). Eur. J. Cancer.

[15] U.S. Department of Health and Human Services Common Terminology Criteria for Adverse Events (CTCAE). (No Title), 2017. https://ctep.cancer.gov/protocoldevelopment/electronic_applications/docs/CTCAE_v5_Quick_Reference_5x7.pdf.

[16] Poplin E., Feng Y., Berlin J., Rothenberg M.L., Hochster H., Mitchell E., Alberts S., O’Dwyer P., Haller D., Catalano P. (2009). Phase III, randomized study of gemcitabine and oxaliplatin versus gemcitabine (fixed-dose rate infu-sion) compared with gemcitabine (30-minute infusion) in patients with pancreatic carcinoma E6201: A trial of the Eastern Cooperative Oncology Group. J. Clin. Oncol..

[17] Small W., Berlin J., Freedman G.M., Lawrence T., Talamonti M.S., Mulcahy M.F., Chakravarthy A.B., Konski A.A., Zalupski M.M., Philip P.A. (2008). Full-Dose Gemcitabine With Concurrent Radiation Therapy in Patients With Nonmetastatic Pancreatic Cancer: A Multicenter Phase II Trial. J. Clin. Oncol..

[18] Murphy J.D., Adusumilli S., Griffith K.A., Ray M.E., Zalupski M.M., Lawrence T.S., Ben-Josef E. (2007). Full-Dose Gemcitabine and Concurrent Radiotherapy for Unresectable Pancreatic Cancer. Int. J. Radiat. Oncol..

[19] Chauffert B., Mornex F., Bonnetain F., Rougier P., Mariette C., Bouché O., Bosset J.F., Aparicio T., Mineur L., Azzedine A. (2008). Phase III trial comparing intensive induction chemoradiotherapy (60 Gy, infusional 5-FU and in-termittent cisplatin) followed by maintenance gemcitabine with gemcitabine alone for locally advanced unresectable pancreatic cancer. Definitive results of the 2000–01 FFCD/SFRO study. Ann. Oncol..

[20] Loehre P.J.L., Feng Y., Cardenes H., Wagner L., Brell J.M., Cella D., Flynn P., Ramanathan R.K., Crane C.H., Alberts S.R. (2011). Gemcitabine Alone Versus Gemcitabine Plus Radiotherapy in Patients With Locally Advanced Pancreatic Cancer: An Eastern Cooperative Oncology Group Trial. J. Clin. Oncol..

[21] Schellenberg D., Kim J., Christman-Skieller C., Chun C.L., Columbo L.A., Ford J.M., Fisher G.A., Kunz P.L., Van Dam J., Quon A. (2011). Single-Fraction Stereotactic Body Radiation Therapy and Sequential Gemcitabine for the Treatment of Locally Advanced Pancreatic Cancer. Int. J. Radiat. Oncol..

[22] Polistina F., Costantin G., Casamassima F., Francescon P., Guglielmi R., Panizzoni G., Febbraro A., Ambrosino G. (2010). Unresectable Locally Advanced Pancreatic Cancer: A Multimodal Treatment Using Neoadjuvant Chemoradiotherapy (Gemcitabine Plus Stereotactic Radiosurgery) and Subsequent Surgical Exploration. Ann. Surg. Oncol..

[23] Chang D.T., Schellenberg D., Shen J., Kim J., Goodman K.A., Fisher G.A., Ford J.M., Desser T., Quon A., Koong A.C. (2009). Stereotactic radiotherapy for unresectable adenocarcinoma of the pancreas. Cancer.

[24] Mahadevan A., Miksad R., Goldstein M., Sullivan R., Bullock A., Buchbinder E., Pleskow D., Sawhney M., Kent T., Vollmer C. (2011). Induction Gemcitabine and Stereotactic Body Radiotherapy for Locally Advanced Nonmetastatic Pancreas Cancer. Int. J. Radiat. Oncol..

[25] Didolkar M.S., Coleman C.W., Brenner M.J., Chu K.U., Olexa N., Stanwyck E., Yu A., Neerchal N., Rabinowitz S. (2010). Image-Guided Stereotactic Radiosurgery for Locally Advanced Pancreatic Adenocarcinoma Results of First 85 Patients. J. Gastrointest. Surg..

[26] Koong A.C., Le Q.T., Ho A., Fong B., Fisher G., Cho C., Ford J., Poen J., Gibbs I.C., Mehta V.K. (2004). Phase I study of stereotactic radiosurgery in patients with locally advanced pancreatic cancer. Int. J. Radiat. Oncol..

[27] Rwigema J.C., Parikh S.D., Heron D.E., Howell M., Zeh H., Moser A.J., Bahary N., Quinn A., Burton S.A. (2010). Stereotactic Body Radiotherapy in the Treatment of Advanced Adenocarcinoma of the Pancreas. Am. J. Clin. Oncol..

[28] Tozzi A., Comito T., Alongi F., Navarria P., Iftode C., Mancosu P., Reggiori G., Clerici E., Rimassa L., Zerbi A. (2013). SBRT in unresectable advanced pancreatic cancer: Preliminary results of a mono-institutional expe-rience. Radiat. Oncol..

[29] Song Y., Yuan Z., Li F., Dong Y., Zhuang H., Wang J., Wang P., Chen H. (2015). Analysis of clinical efficacy of CyberKnife^®^ treatment for locally advanced pancreatic cancer. OncoTargets Ther..

